# Gene expression profiles of bovine uninucleate trophoblast cells and trophoblast giant cells: a data note

**DOI:** 10.1186/s13104-020-04964-z

**Published:** 2020-02-27

**Authors:** Marina Polei, Juliane Günther, Dirk Koczan, Rainer Fürbass

**Affiliations:** 1 Institute of Reproductive Biology, Leibniz Institute for Farm Animal Biology (FBN), 18196 Dummerstorf, Germany; 2 Institute for Genome Biology, Leibniz Institute for Farm Animal Biology (FBN), 18196 Dummerstorf, Germany; 3 Institute of Immunology, University of Rostock, 18057 Rostock, Germany

**Keywords:** Placenta, Placentome, UTC, TGC, Cell differentiation

## Abstract

**Objectives:**

In the bovine placenta, intimate fetomaternal contact is restricted to placentomes. Within the placentomes fetal chorionic villi interdigitate with corresponding maternal caruncular crypts. The trophoblast epithelium covering the chorionic villi consists of 80% uninucleate trophoblast cells (UTCs) and 20% trophoblast giant cells (TGCs). TGCs migrate toward the endometrium and fuse with endometrial cells to form short-lived fetomaternal hybrid cells. Thereby the TGCs transport molecules of fetal origin across the placental barrier into the maternal compartment. The UTC/TGC ratio is constant during pregnancy because UTCs can differentiate into new TGCs to replace spent TGCs. However, our understanding of this differentiation process was sparse. Therefore, we collected the data to study the gene expression profiles in UTCs and TGCs and to identify differently expressed genes between the two trophoblast cell populations. Using Gene Ontology analysis, we wanted to identify biological processes and pathways that play an important role in the differentiation of UTCs into TGCs.

**Data description:**

Bovine placentas were from days 118 to 130 of gestation. We obtained virtually pure UTCs and TGCs using a fluorescence-activated cell sorting (FACS) method. Total RNA was extracted from the UTC and TGC isolates, labeled and hybridized to Affymetrix Bovine Gene 1.0 ST Arrays.

## Objective

In the bovine placenta, intimate fetomaternal contact is restricted to discrete mushroom-shaped structures termed placentomes. Within the placentomes widely ramified fetal chorionic villi interdigitate with corresponding maternal caruncular crypts. The trophoblast epithelium covering the chorionic villi consists of two cell types: 80% uninucleate trophoblast cells (UTCs) and 20% trophoblast giant cells (TGCs). The TGCs are an important source of estrogens that act on the maternal endometrium, but also on the trophoblast itself. In addition, the cytoplasm of TGCs includes numerous granules containing placental lactogen (PL) and pregnancy-associated glycoproteins (PAGs).The TGCs are capable of migrating toward the endometrium and fuse with endometrial cells to form short-lived fetomaternal hybrid cells. Thereby the TGCs transport molecules of fetal origin, including PL and PAGs, across the placental barrier into the maternal compartment. The UTC/TGC ratio is almost constant during pregnancy because UTCs can differentiate into new TGCs to replace spent TGCs. This ongoing regeneration of TGCs is essential for maintaining pregnancy but our understanding of this differentiation process was sparse. Therefore, we performed this first microarray study of virtually pure bovine UTCs and TGCs to analyze their gene expression profiles and identify differentially expressed genes between the two trophoblast cell populations. Using Gene Ontology analysis, we wanted to identify biological processes and pathways that play an important role in the differentiation of UTCs into TGCs [1].

## Data description

Four bovine placentas of gestation days 118 to 130 were collected at a local slaughterhouse and processed immediately. The procedure for isolating the trophoblast cells is described in detail in Polei et al. [2]. Briefly, 25 to 35 placentomes per animal were dissected, and the cotyledons were manually separated from the caruncles. For tissue disintegration, the cotyledonal villi were digested with collagenase (catalog number 17456; Serva, Heidelberg, Germany). The resulting trophoblast cell suspensions were loaded on discontinuous Percoll gradients with 1.03, 1.04, 1.05, 1.06 g Percoll/ml (L6143, 1.124 g/ml; Biochrom, Berlin, Germany) and centrifuged at 1200*g* for 20 min. The trophoblast cells obtained from interphases 1.03/1.04 and 1.04/1.05 were pooled, stained with Hoechst 33342 (14533; Sigma-Aldrich, Traufkirchen, Germany) and sorted with a MoFlo-XDP cell sorter (Beckman Coulter, Krefeld, Germany). Scatter plots of height versus area of Hoechst signals were used for duplex elimination. Fluorescence histograms of single cells were used to discriminate diploid UTCs and poliploid TGCs. Analysis by microscopy demonstrated virtually pure UTC and TGC isolates. Experimental procedures for the microarray analyses are described in detail by Polei et al. [1] and briefly summarized below. UTCs and TGCs from three of the four placentas (from animals 2, 3 and 4 [2]) yielded sufficient amounts of RNA for microarray analyses. Total RNA preparation with the NucleoSpin RNA II Kit (740955; Macherey–Nagel, Düren, Germany) included removal of genomic DNA with RNAse-free recombinant DNAse. Analyses of RNA quality in a 2100 Bioanalyzer instrument using the RNA 6000 Pico Kit and 2100 Expert Software (Agilent Technologies, Santa Clara, CA, USA) yielded RNA integrity numbers between 7.2 and 8.8. To obtain labeled hybridization probes, single strand DNA (ssDNA) was generated from the RNA of each cell sample using the Ambion WT Expression Kit (4411973; Thermo Fisher Scientific, Waltham, MA, USA) and the ssDNA was then fragmented and labeled using the Affymetrix Gene Chip WT Terminal Labeling and Hybridization Kit (Affymetrix, Santa Clara, CA, USA). Hybridization of the labeled probes to Affymetrix Bovine Gene 1.0 ST Arrays was performed in an Affymetrix Gene Chip hybridization oven for 16 h at 45 ℃. Scanning of the microarrays at a resolution of 0.7 micron was performed with an Affymetrix Genechip Scanner 3000 7G. The Biometric Research Branch (BRB) Array Tools version 4.4.1 [3] were used for the analysis of the microarray data and the GC Robust Multi-Array Average (GC RMA) algorithm [4] for background correction and normalization of the expression values. The calculated signal intensity values of the microarray scans are shown in the Table 1, Data files 1 to 6.

**Table 1:** Overview of data files

Label	Name of data file/data set	File types (file extension)	Data repository and identifier (DOI or accession number)
Data file 1	Primary bovine trophoblast giant cells from cow 2	Affymetrix CEL file (.cel)	GSM3466983 [5]
Data file 2	Primary bovine uninucleate trophoblast cells from cow 2	Affymetrix CEL file (.cel)	GSM3466984 [6]
Data file 3	Primary bovine trophoblast giant cells from cow 3	Affymetrix CEL file (.cel)	GSM3466985 [7]
Data file 4	Primary bovine uninucleate trophoblast cells from cow 3	Affymetrix CEL file (.cel)	GSM3466986 [8]
Data file 5	Primary bovine trophoblast giant cells from cow 4	Affymetrix CEL file (.cel)	GSM3466987 [9]
Data file 6	Primary bovine uninucleate trophoblast cells from cow 4	Affymetrix CEL file (.cel)	GSM3466988 [10]

## Limitations

- Pregnancy lasts 270 days in cattle. Since trophoblast cells were obtained from bovine placentas from 118 to 130 days of pregnancy, the data may only be valid for this period.
- We could analyze UTCs and TGCs from only three placentas.

## Data Availability

The data described in this Data note can be freely and openly accessed on the Gene Expression Omnibus (GEO) database under accession number GSE122474 [11]. Please see Table 1 and references [1–11] for details and links to the data.

## Abbreviations

FACS: Fluorescence activated cell sorting
UTC: Uninucleate trophoblast cell
TGC: Trophoblast giant cell
